# Gains in awareness, ownership and use of insecticide-treated nets in Nigeria, Senegal, Uganda and Zambia

**DOI:** 10.1186/1475-2875-7-153

**Published:** 2008-08-07

**Authors:** Carol A Baume, M Celeste Marin

**Affiliations:** 1 Academy for Educational Development, 1825 Connecticut Avenue NW, Washington, DC, 20009, USA

## Abstract

**Background:**

In April 2000, the Roll Back Malaria (RBM) "Abuja Summit" set a target of having at least 60% of pregnant women and children under five use insecticide-treated nets (ITNs). Thereafter, programmes were implemented to create demand, reduce taxes and tariffs, spur the commercial market, and reach vulnerable populations with subsidized ITNs. Using national ITN monitoring data from the USAID-sponsored AED/NetMark project, this article examines the extent to which these activities were successful in increasing awareness, ownership, and use of nets and ITNs.

**Methods:**

A series of surveys with standardized sampling and measurement methods was used to compare four countries at two points in time. Surveys were conducted in 2000 and again in 2004 (Nigeria, Senegal, Zambia) or 2006 (Uganda). They contained questions permitting classification of each net as untreated, ever-treated or currently-treated (an ITN). Household members as well as nets owned were enumerated so that households, household members, and nets could be used as units of analysis. Several measures of net/ITN ownership, plus RBM ITN use indicators, were calculated. The results show the impact of ITN activities before the launch of massive free net distribution programmes.

**Results:**

In 2000, treated nets were just being introduced to the public, but four to six years later the awareness of ITNs was nearly universal in all countries but Nigeria, where awareness increased from 7% to 60%. By any measure, there were large increases in ownership of nets, especially treated nets, in all countries. All countries but Nigeria made commensurate gains in the proportion of under-fives sleeping under a net/ITN, and in all countries the proportion of pregnant women sleeping under a net/ITN increased greatly.

**Conclusion:**

A mix of demand creation, a strengthened commercial sector, reduced taxes and tariffs, and programmes making ITNs available at reduced prices resulted in impressive gains in awareness, ownership, and use of nets and ITNs in Nigeria, Senegal, Zambia, and Uganda between 2000 and 2004–2006. None of the countries reached the ambitious Abuja targets for ITN use, but they made substantial progress towards them.

## Background

One of the most effective tools for malaria prevention is the insecticide-treated mosquito net (ITN). Consistent use of ITNs can reduce malaria transmission by up to 90% [1] and avert as much as 44% of all-cause mortality among children under five [2,3]. Use of ITNs among pregnant women is associated with lower prevalence of malaria infection, fewer premature births and significant reductions in all-cause maternal anaemia [4-7]. Even untreated nets provide protection, though ITNs are approximately twice as effective as untreated nets [8,9].

The Roll Back Malaria (RBM) African Summit held in Abuja, Nigeria on April 25, 2000, set a target of having at least 60% of children under five years of age and 60% of pregnant women use ITNs. Thereafter, malaria-prone countries undertook some combination of education, demand creation, reduction of taxes and tariffs on ITNs, commercial ITN market development, and programmes to reach the most vulnerable populations with subsidized ITNs. To what extent were these activities successful at generating ITN ownership and use?

The data for addressing these questions come from four African countries in which large-scale household surveys were conducted in 2000 and again in 2004 (Nigeria, Senegal and Zambia) or 2006 (Uganda). The data were collected by NetMark, an AED (Academy for Educational Development) project funded by the United States Agency for International Development (USAID). NetMark's goal is to reduce the burden of malaria by developing a sustainable commercial supply of ITNs, and to ensure that economics is never an obstacle to ownership by implementing complementary targeted subsidy programmes that combine discount vouchers and fee distribution. Its partners include over 40 national and international insecticide and net manufacturers, product distributors, and advertising companies. As part of its research activities, NetMark conducts household surveys as well as market impact studies and other types of research. The surveys intend to measure changes in the country as a whole that result from activities by a number of actors, although all are countries in which NetMark strengthened the commercial market to provide a sustainable supply of ITNs and played the lead role in airing mass media for demand creation and in working toward reduction or elimination of government taxes and tariffs on ITNs. In addition to the four countries reported on in this paper, NetMark has carried out surveys in Mozambique, Mali, Ghana, and Ethiopia. Data from those countries are not included since the surveys were administered once; we include here only countries with follow-up surveys that permit measurement of change over time.

This paper reports on progress since 2000 in Nigeria, Senegal, Uganda, and Zambia in key indicators of change: awareness of ITNs, ownership of nets and ITNs, and the proportion of children under five and pregnant women sleeping under nets and ITNs. Although the Abuja targets refer only to treated nets, this study also reports on untreated nets and ever-treated nets, since they also reduce malaria transmission. By including data on all nets, the extent to which acceptance of bed nets in general has increased can be shown. Furthermore, data measuring untreated as well as treated nets helps ITN promotion programmes determine the extent to which emphasis needs to be on net acquisition as well as on treatment of nets already owned.

## Methods

### Sample

The surveys used multi-stage stratified sampling. In each country, the sample was drawn from five primary sites, including the capital or main commercial city. The sites were located in malarious areas and purposively selected to reflect the geo-ethnic diversity of the country. Each site includes both urban and rural households: 40% of households were selected from the city and 60% from up to 200 kilometers from the city. The 40:60 ratio approximates the urban-rural distribution in the countries surveyed, with the exception of Uganda, where approximately 12% of the population is urban. The Uganda data have been weighted to take this urban-rural ratio into account. In each site, 20–40 sampling points were selected so as to result in 10 households per sampling point. The sampling points were drawn from census or other available enumeration lists.

Respondents were women of reproductive age (15–49) who were mothers or guardians of children under five years of age. These inclusion criteria were selected to maximize the number of pregnant women and children under five in the sample, the basis for the key RBM indicators. Informed consent was obtained from all respondents by having the interviewer read the consent statement, ask the respondent if she wished to participate, and sign that the consent form had been read and agreed to.

The sample size was 1,000–2,000 households per country per wave. Table 1 shows the sites, number of households and family members, and numbers of nets in each wave and country. Some analyses are based on households, some on family members, and some on nets.

**Table 1:** Sample sizes: Number of households, family members, and nets, by country and year

Country and sites (site = urban + rural surroundings)	2000			2004		
	
	#HH	#people	#nets	#HH	#people	#nets
**Nigeria**: Lagos, Ibadan, Kano Maiduguri, Nsukka	1000	4802	120	2000	10791	533
**Senegal**: Dakar, Thies, St. Louis, Kaolack, Tambacounda	1000	7770	336	2000	17433	1122
**Uganda**: Kampala, Masaka, Mbarara , Soroti, Hoima	1000	4742	340	*2122	*10747	*1079
**Zambia**: Lusaka, Choma, Kaoma, Kitwe Mansa	1000	5853	265	2000	11608	999

*data collected in 2006

The data from these surveys are structured to provide a valid measure of change over time. The same sites, the same sampling procedure within sites, and the same indicators were used at both points in time. The fieldwork was conducted at the same time of year to control for seasonal variation. In all countries except Zambia, the data were collected during the rainy season when malaria transmission is highest. (In Zambia, the baseline data was needed before the rainy season started.)

It should be noted that levels of net ownership in this sample are likely to be higher than those derived from a national random sample, for a number of reasons. The sample included only households with children under five, and households with young children are more likely than those without to own a net. Furthermore, the sample was drawn only from areas where malaria is a problem, and net ownership will be far higher in those areas than in non-malarious areas of a country. Another reason why results may differ from those of a national random sample is that the sample was divided evenly among the sites, even though population sizes in the sites differ. None of these factors affects the validity of the data as a measure of the magnitude of change in the sites sampled, the primary purpose of this paper.

### Definitions of nets by type and treatment status

In net-owning households, a series of questions was asked to allow as accurate as possible determination of the treatment status of each net owned: never treated, ever treated, or currently treated. Questions included how long ago the net was acquired, whether the net was pretreated by the manufacturer or treated by someone (for example a health worker); whether the net came packaged with an insecticide treatment kit; whether the net has ever been treated with insecticide to kill or repel mosquitoes; and if so, how long ago the net was treated. Other questions that could help confirm treatment status were also asked: whether the net was treated by someone in the household or taken somewhere else such as a health center; if the net was manufactured or tailor-made; and what the brand, size, and colour and source of the net was. Nets distributed by projects or donors are usually available in one size and colour, so these characteristics can help identify the type of net owned – for example whether it is a LLIN, or long lasting insecticide-treated net. By 2006, when the Uganda data were collected, most nets had tags on them with the manufacturer or batch number, which was recorded to further confirm net type and treatment status.

The questionnaire asked separately about mosquito nets that can be hung and baby nets that are placed over an infant. The net definitions used in this paper are as follows:

• *Net*: a net that can be hung for use while sleeping regardless of whether it has ever been treated

• *Ever-treated net*: a net that has ever been treated, either when acquired (pre-treated) or since acquired, regardless of when the treatment was put on the net

• *ITN or currently-treated net*: a net that is a "permanently treated" or long lasting insecticide-treated net (a LLIN), or is pre-treated and has been purchased within the last 12 months, or has had insecticide put on it up to and including the last 12 months. This is the Roll Back Malaria (RBM) definition of an ITN.

• *Baby net*: a small umbrella-like net with a built-in frame that is not hung but is placed over an infant. It is often used to keep flies off a sleeping infant during the day, but can also be used at night. Baby nets are rarely treated, and the umbrella frame precludes dipping the netting in an insecticide solution.

The study distinguishes between nets of varying treatment status because different profiles of net coverage by treatment status have different implications for the direction that an ITN promotion programme should take. For example, if there has been a large increase in net ownership but few nets have ever been treated, this implies acceptance of the concept of nets but a problem in access to or acceptability of treated nets. If there were a large number of "never-treated" or even "ever-treated" nets, but few currently treated ones, there would need to be a focus on getting people to re-treat nets or, given the newer technology of LLINs, to acquire a net with a long-lasting treatment on it.

Household members were enumerated and linked to a specific net to enable calculation of the percent sleeping under any net (whether or not it was treated) and the percent under a currently treated net. A file with the net as the unit of the analysis was created to permit analyses on the population of nets in the sample. A file with each household member as the unit of analysis was also generated in order to calculate proportions of household members sleeping under the net.

## Results

The following reports on changes in awareness, ownership, and use of nets and ITNs between 2000 and the follow-up survey. Table 2 summarizes the results reported in this section. With one exception noted in the text, all changes were statistically significant at the p < 0.05 level, so we have not reported each separately.

**Table 2:** Awareness, ownership, and use of nets and ITNs, by country and year

	**NIGERIA**		**SENEGAL**		**ZAMBIA**		**UGANDA**	
	
	**2000**	**2004**	**2000**	**2004**	**2000**	**2004**	**2000**	**2006**
**% respondents aware of treated nets**	7.3	60.3	70.0	97.3	50.7	88.3	23.2	97.6

**N**	1000	2000	1000	2000	1000	2000	1000	2122

**% HH owning a net**	12.0	26.7	33.6	56.1	26.5	50.0	30.4	44.4
**Average number of nets owned**	1.3	1.7	2.1	2.8	1.4	1.8	1.6	1.6
**% HH owning ever-treated net**	0.0	9.8	11.0	42.8	9.5	40.3	2.3	24.1
**% HH owning currently-treated net (ITN)**	0.0	8.9	8.2	38.7	5.5	34.6	1.4	20.9
**% HH owning a baby net (non-hanging)**	*	39.9	*	9.5	*	1.4	*	1.0

**N**	1000	2000	1000	2000	1000	2000	1000	2122

**% of nets never treated**	99.4	70.0	69.8	49.0	64.2	20.0	92.3	52.0
**% nets ever treated**	0.6	30.5	30.2	72.9	35.8	80.3	7.7	48.1
**% ITNs (treated within 12 months)**	0.0	27.1	23.4	65.0	21.5	65.6	4.6	40.4

**N**	159	856	649	2579	363	1734	586	1734

**% children < 5 slept under a net last night**	8.8	§10.3	17.7	35.4	11.9	24.6	20.5	29.6
**% children < 5 slept under a net or a baby net last night**	*	17.9	*	37.6	*	24.7	*	*
**% children < 5 slept under an ITN last night**	0.0	3.3	4.6	23.9	2.4	16.9	1.2	13.5

**N**	1402	3054	1811	4116	1470	2862	1361	3124

**% pregnant women sleeping under a net**	7.3	14.1	21.7	41.7	3.9	21.5	17.3	29.4
**% pregnant women sleeping under an ITN**	0	4.4	5.0	31.0	0	13.5	1.3	13.3

**N**	96	249	120	290	76	237	130	362

* Data not collected

§ This is the only increase that is not statistically significant at the p < 0.05 level; in this case p = 0.07

### Awareness of ITNs

**Figure 1:**
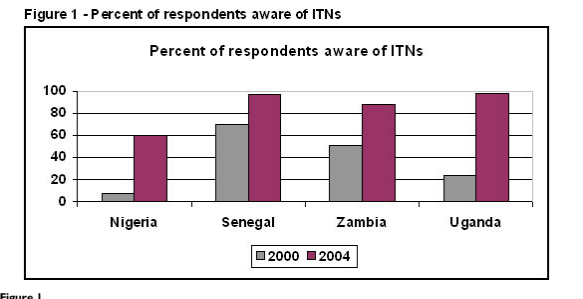
**Percent of respondents aware of ITNs**. Awareness of ITNs in 2000 is shown in the lighter bar on the left and awareness in 2004 is shown in the darker bar on the right.

In 2000, treated nets had just been introduced to the public. In some countries such as Senegal untreated nets made from various fabrics had long been used; in other countries such as Zambia few families had ever used any kind of bed net. As the indicator for "awareness", respondents were asked if they had ever heard of mosquito nets that had been dipped or soaked in insecticide to kill or repel mosquitoes (Figure 1). Only 7% of Nigerians had even heard of treated nets in 2000. In Uganda 23% had, in Zambia 51%, and in Senegal 70% had. By 2004–2006, awareness of ITNs was nearly universal in all countries except Nigeria, though awareness there was very low to start with and jumped from 7% to 60% between 2000 and 2004.

### Household ownership of nets and ITNs

Net ownership was measured in several ways, by calculating:

▪ the percent of households owning a bed net (whether treated or not)

▪ the percent of households owning an ITN, an ever-treated net, and a never-treated net

▪ the percent of nets owned that had been treated

▪ the average number of nets owned by net-owning households.

The figures reported here include only hanging bed nets; they do not include baby nets.

**Figure 2:**
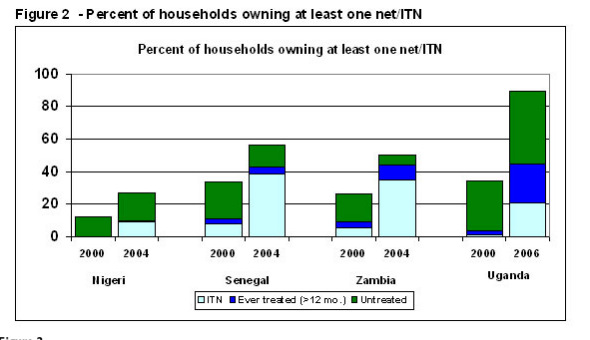
**Percent of households owning at least one net/ITN**. The bottom segment shows households owning at least one ITN (a net treated within the past year), the middle segment shows the percent of households owning at least one net that was treated more than 12 months ago, and the top segment shows the percent owning an untreated net.

By any measure, there were large increases in the ownership of nets, and especially treated nets, in all countries (Figure 2). The countries with the lowest percent of households owning a net to begin with doubled their net coverage, and those with higher initial coverage made very large gains. For example, between 2000 and 2004, the percent of households owning a hanging net (treated or untreated) rose from 12% to 27% in Nigeria and from 34% to 56% in Senegal. For ever-treated nets, the rise was from 0% to 10% in Nigeria and from 11% to 43% in Senegal. There were especially large increases in the percent of households owning an ITN – from 8% to 39% in Senegal between 2000 and 2004, and from 1% to 21% in Uganda between 2000 and 2006.

In addition to the increase in the percent of households owning a net or ITN, the average number of nets owned by net-owing households increased in three of the countries – from 1.3 to 1.7 in Nigeria; from 2.1 to 2.8 in Senegal; from 1.4 to 1.8 in Zambia. Therefore, the number of nets in those countries increased tremendously between 2000 and 2004 – both because many more households owned nets, and because households tended to acquire more than one net. In Uganda, the percent of net-owning households increased considerably between 2000 and 2006, but the average number of nets owned per household remained the same at 1.6.

### Proportion of nets that have been treated

**Figure 3:**
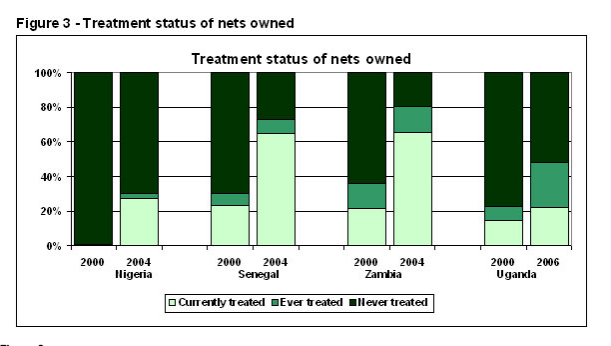
**Treatments status of nets owned**. The bottom segment shows the percent of all nets owned that were ITNs, the middle segment shows the percent of nets owned that were treated more than 12 months ago, and the top segment shows the percent that were never treated.

Using nets as the unit of analysis, calculations of the proportion of nets falling into each treatment category show an especially high increase in currently-treated nets between the two survey periods: from 0% to 27% in Nigeria; from 23% to 65% in Senegal, from 22% to 66% in Zambia, and from 5% to 40% in Uganda (Figure 3).

The increase was due both to increases in nets that were "pre-treated" (already had treatment on them when acquired) and "post-treated" nets (had treatment put on them after they were acquired). For example, the percent of nets that were pre-treated rose from 0% to 20% in Nigeria; from 18% to 65% in Senegal, from 28% to 62% in Zambia, and from 5% to 29% in Uganda. Substantial increases in the percent of nets treated since acquired were also seen: from 0% to 18% in Nigeria; from 15% to 26% in Senegal; from 16% to 42% in Zambia; and, from 6% to 26% in Uganda.

### Percent of vulnerable groups sleeping under nets/ITNs

A crucial goal of malaria control programmes is to achieve consistent ITN use by children under five and pregnant women. Although it is beneficial for any household member to sleep under a net, it is particularly important for those most vulnerable to severe malaria to do so. The following reports the proportion of children under five and pregnant women in all households sleeping under nets and ITNs, thus showing the status of the sample with regard to the Abuja targets.

Use by vulnerable groups requires that a household own a net, and that the most vulnerable groups be given priority for sleeping under the net. Therefore, the proportion of a population under a net/ITN is limited by and to some extent reflects net/ITN ownership rates. However, usage rates are not an exact mirror of ownership rates because usage is also affected by intra-household factors such as the extent to which nets are used at all, family sleeping patterns, and who in the household uses the net [10]. Further, net use varies by season and is likely to be highest in mid-end rainy season, when mosquito density and malaria transmission is highest. In all countries except Zambia, the data were collected at the onset or middle of the rainy season. In Zambia the data were collected before the rainy season, so net use figures for Zambia are likely to be lower than they would be if data were collected during the rainy season.

**Figure 4:**
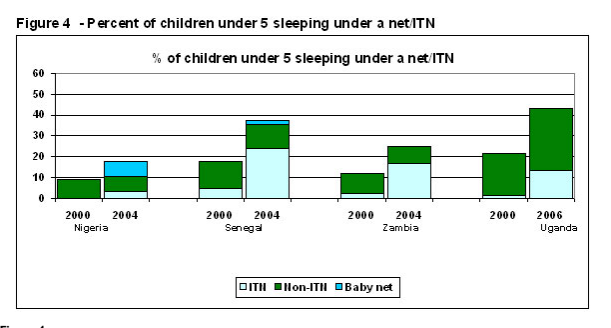
**Percent of children under five sleeping under a net/ITN**. The bottom segment shows the percent of children under five that slept under an ITN, the middle segment shows the percent of children that slept under a net that was not an ITN (either never treated or treated 12 more than months ago), and the top segment shows the percent that slept under a baby net.

Between 2000 and 2004, the percent of children under five who slept under a net (a hanging net, whether treated or untreated) the prior night doubled in Senegal (from 18% to 35%) and Zambia (from 12% to 25%) (Figure 4). Between 2000 and 2006 in Uganda, the percent rose from 21% to 28%. The relative increase in use of ITNs for under-fives was much larger: from 5% to 25% in Senegal, from 2% to 17% in Zambia, and from 1% to 14% in Uganda. However, in Nigeria, the increase was very small: from 8.8% to 10.3% sleeping under a net, and from 0% to 3.3% sleeping under an ITN. The proportion of Nigerian under-fives sleeping under a net was the only gain measured in this study that did not attain statistical significance at the p < 0.05 threshold; it was p = 0.07. Even though net/ITN ownership did increase considerably in Nigeria in the period between surveys, a much larger proportion of those nets went unused in 2004 than in 2000. The picture for Nigeria does improve if infants sleeping under a baby net are included. Baby nets are common in Nigeria but not in the three other countries. Baby net data were not collected in 2000, but in 2004, if infants sleeping under a baby net are included, the percent of under-fives in Nigeria sleeping under some kind of net the prior night was18%.

**Figure 5:**
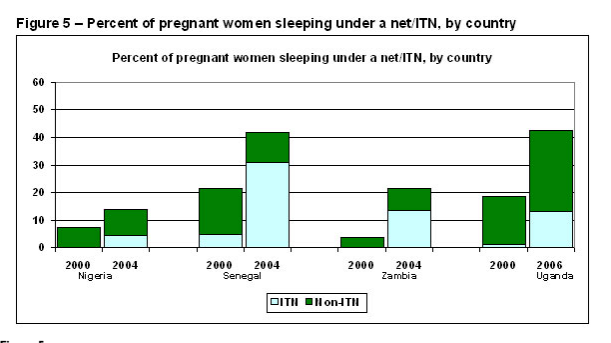
**Percent of pregnant women sleeping under a net/ITN**. The bottom segment shows the percent of pregnant women that slept under an ITN and the top segment shows the percent of pregnant women that slept under a net that was not an ITN.

All countries achieved large increases in the percent of pregnant women sleeping under a net, rising from 7% to 14% in Nigeria, from 22% to 42% in Senegal, from 4% to 22% in Zambia, and from 17% to 30% in Uganda (Figure 5). The use of ITNs by pregnant women showed steeper proportional gains in some countries, increasing from 5% to 31% in Senegal, from 0% to 14% in Zambia, and from 1% to 13% in Uganda. The gain in Nigeria was smaller: from 0% to 5%.

## Discussion and Conclusion

The body of data reported on here is particularly useful in that it employs standardized sampling and measurement methods to compare several countries, each at two points in time, to examine changes in ITN-related variables in detail. The questionnaire contains a set of questions that permits classification of a given net as untreated, ever-treated and currently-treated, using the RBM definition of an ITN. RBM has defined an ITN as one that has been treated within the last 12 months, but definitions of an ITN across studies vary. Some surveys classify a net as an ITN based only on the response to a question asking if the net was ever treated with a product to kill mosquitoes, thereby classifying ever-treated nets as ITNs [11]. Some studies simply ask if the household owns an ITN, but the respondent may not be well informed about what an ITN is and whether her net qualifies as one. Use of the RBM definition is important in order to give precise meaning to data concerning ITNs. The data presented here are based on rigorous definitions of types of nets and of treatment status.

The data document considerable progress between 2000 and 2004–2006 by organizations active in promoting ITNs to combat malaria. The main activities employed during that period – increasing the role of the private sector to procure, market, and distribute ITNs; reducing or eliminating taxes and tariffs; making nets affordable through subsidies or vouchers; creating demand through mass media, road shows, and interpersonal communication with health providers – resulted in often dramatic increases in ownership and use of nets and ITNs in four African countries with very different cultural and socio-political contexts. Not only has the percent of households owning any net approximately doubled, but the number of nets owned per net-owning household has increased. In 2000, in most countries a minority of respondents had even heard of nets treated with insecticide, and extremely few households owned them. Since that time, the picture has changed radically. Generally there has been a tripling in the proportion of nets that are ITNs. Nonetheless, the data show that at least one-third of nets owned are not currently treated. These untreated nets could be easily converted to ITNs if programmes facilitated or promoted net treatment, thereby rapidly increasing ITN coverage.

Expanded net ownership between 2000 and 2004 resulted in a 100% increase in the percent of children under five sleeping under a net in Senegal and Zambia; and expansion of net ownership between 2000 and 2006 in Uganda resulted in a 50% increase in the percent of young children under a net. However, in Nigeria, even though net ownership doubled, the increase in under-fives protected by a net was negligible since 44% of nets went unused – far higher than the 9% that went unused in 2000. The reason for the increase in unused nets in Nigeria warrants further study.

The acceptance of ITNs for use by pregnant women rose substantially in all countries. After consumer research showed that fear of the chemical on treated nets was a barrier to use of ITNs by pregnant women, messages were developed to counter this incorrect belief. Data from other parts of the survey not reported here show that the levels of fear about the safety of the insecticide for pregnant women have dropped since 2000. For example, in Zambia, the proportion of survey respondents – all women of reproductive age – who spontaneously said that the chemical on the net could be dangerous to a pregnant woman or her foetus dropped from 27% to 7% between 2000 and 2004.

The timing of these surveys enhances their utility, since it coincided with shifts in the intensity of ITN promotion and in the focus of ITN distribution methods. The baselines were conducted before there was much promotion of ITNs (in 2000), and the follow-up surveys were conducted before the launch of massive free net programmes. Only a small percentage of nets were free in 2004 (Nigeria 11%, Senegal 3%, Zambia 3%) and 2006 (Uganda 9%). Surveys taken now – during or following major free net distribution efforts – should find even sharper increases in net ownership [11], and it will be useful to track trends in net retention, use, and replacement as programme approaches change. The tremendous gains achieved in all four countries before the implementation of large-scale free net programmes suggest that free net distribution should complement rather than replace other approaches. Targeting free nets to poorer families while giving those with more resources the option to buy a net in the shape and color they prefer should result in optimal ownership levels. Given that, with sufficient demand, the commercial supply system requires little if any donor support for its survival, it is particularly important not to undermine this system.

The Abuja goal of having 60% of children under five and pregnant women sleep under an ITN was ambitious, especially for those countries with initially low net ownership. Although Abuja targets had not been reached in the countries surveyed, the progress made in ITN awareness, ownership, and use was substantial and notable.

## Authors' contributions

Dr. CB has been Principal Investigator for the surveys since their inception in 2000, and drafted the article. Ms. MCM managed the data sets and conducted the data analysis.
